# Supplementary material for: Oxacillin-Supplemented Mueller-Hinton Agar for In Vitro Inhibition of Ambler Class C β-Lactamases in Enterobacterales
Source: Antibiotics (Basel). 2025 Jun 18;14(6):616. doi: 10.3390/antibiotics14060616 (PMC12189709; doi:10.3390/antibiotics14060616)
Supplement: Supplementary file 1 [file antibiotics-14-00616-s001.zip › antibiotics-3691267-supplementary.pdf]

Supplementary tables and figures

Oxacillin-Supplemented Mueller-Hinton Agar for In Vitro Inhibition of Ambler Class C β-Lactamases in Enterobacterales

Edgar-Costin Chelaru [1,2], Andrei-Alexandru Muntean [1,2,*], Mădălina-Maria Muntean [1], Mihai-Octav Hogea [1], Costin-Ștefan Caracoti [1,2], Bogdan-Florin Ciomaga [1], Thierry Naas [3,4,5] and Mircea Ioan Popa [1,2]

1 Discipline of Microbiology II, Department 2, Faculty of Medicine, Carol Davila University of Medicine and Pharmacy, 020021 Bucharest, Romania; edgar-costin.chelaru@drd.umfcd.ro (E.-C.C.); madalina.muntean@umfcd.ro (M.-M.M.); mihai-octav.hogea@drd.umfcd.ro (M.-O.H.); costin-stefan.caracoti@drd.umfcd.ro (C.-Ș.C.); bogdan-florin.ciomaga@drd.umfcd.ro (B.-F.C.); mircea.ioan.popa@umfcd.ro (M.I.P.)

2 Cantacuzino National Military Medical Institute for Research and Development, 050096 Bucharest, Romania

3 Team Resist UMR1184 Immunology of Viral, Auto-Immune, Hematological and Bacterial Diseases (IMVA-HB), INSERM, Faculty of Medicine, CEA, LabEx LERMIT, Université Paris-Saclay, 94270 Le Kremlin-Bicêtre, France; thierry.naas@aphp.fr

4 Associated French National Reference Center for Antibiotic Resistance: Carbapenemase-Producing Enterobacteriaceae, 94270 Le Kremlin-Bicêtre, France

5 Bacteriology-Hygiene Unit, Bicêtre Hospital, Assistance Publique-Hôpitaux de Paris (AP-HP), 94270 Le Kremlin-Bicêtre, France

* Correspondence: alexandru.muntean@umfcd.ro

ATTACHED AS SEPARATE .XLSX FILE

Table S1. Detailed results for all bacterial strains and antibiotics tested on MHO and MHC250.

PASE = penicillinase, BSPASE = broad-spectrum PASE, ESBL = extended spectrum beta-lactamase, CASE = cephalosporinase, HCASE = hyper-cephalosporinase, NA = not applicable, MHA = Mueller-Hinton Agar, MHC250 = Mueller-Hinton Agar supplemented with 250 mg/L cloxacillin, MHO250 / MHO300 / MHO350 = Mueller-Hinton Agar supplemented with 250 / 300 / 350 mg/L oxacillin, ATM = aztreonam (30 µg), CAZ = ceftazidime (10 µg), CTX = cefotaxime (5 µg), ETP = ertapenem (10 µg), FOX = cefoxitin (30 µg), TIC = ticarcillin (75 µg), TCC = ticarcillin/clavulanic acid (75/10 µg).

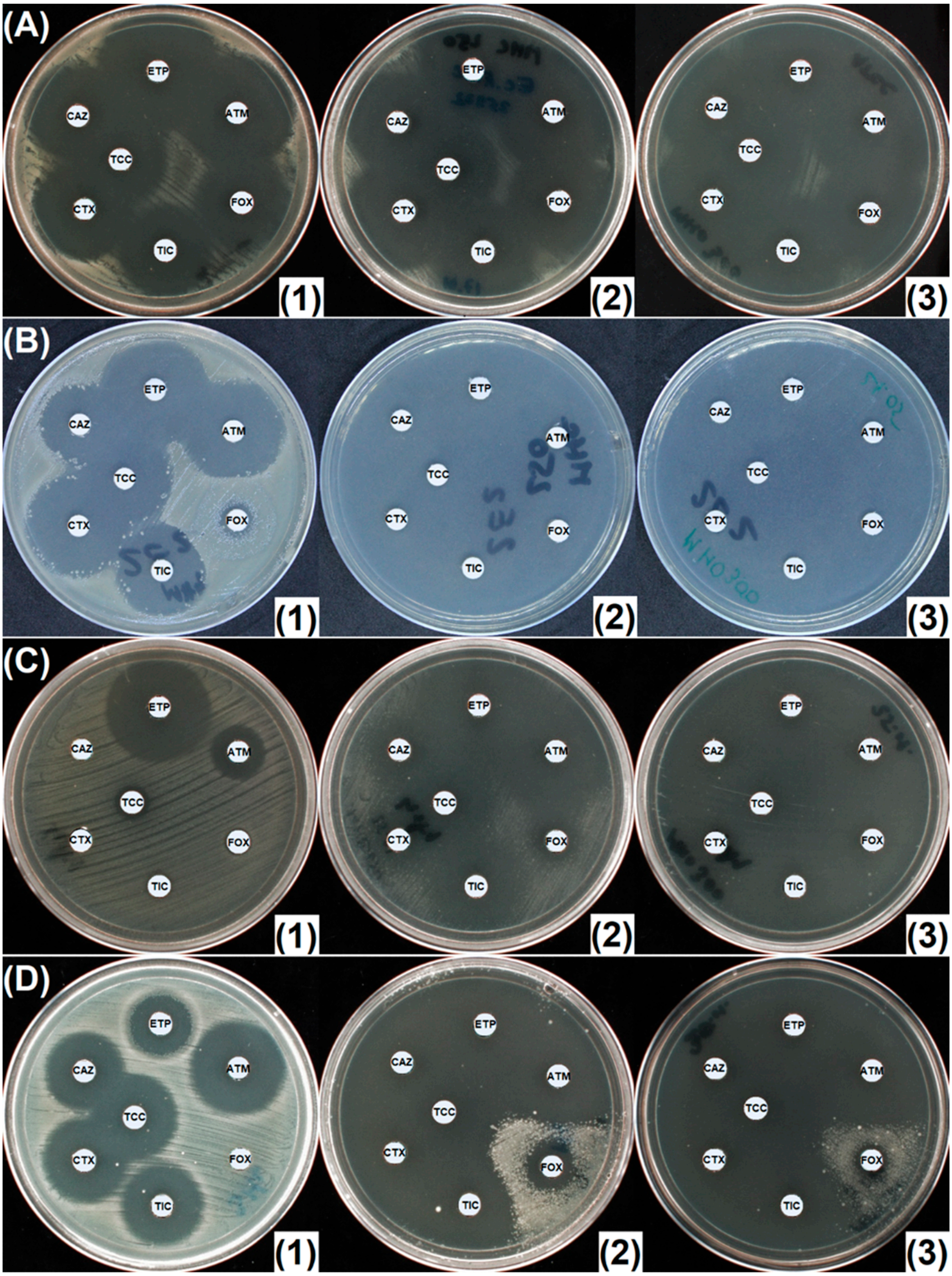

Figure S1. Effect of antibiotics on growth of some Enterobacterales isolates. (A) *E. coli* ATCC 25922; (B): DHA-1 producing *E. coli;* and (C) AmpC hyper-producing *H. alvei.*; (D) NmcA producing *E. cloacae.*

(1) Mueller-Hinton Agar (MHA); (2) Mueller-Hinton Agar supplemented with 250 mg/L cloxacillin (MHC250); (3) Mueller-Hinton Agar supplemented with 300 mg/L oxacillin

(MHO300). ATM: aztreonam (30 µg); CAZ: ceftazidime (10 µg); CTX: cefotaxime (5 µg); ETP: ertapenem (10 µg); FOX: cefoxitin (30 µg); TIC: ticarcillin (75 µg); TTC: ticarcillin/clavulanic acid (75/10 µg).

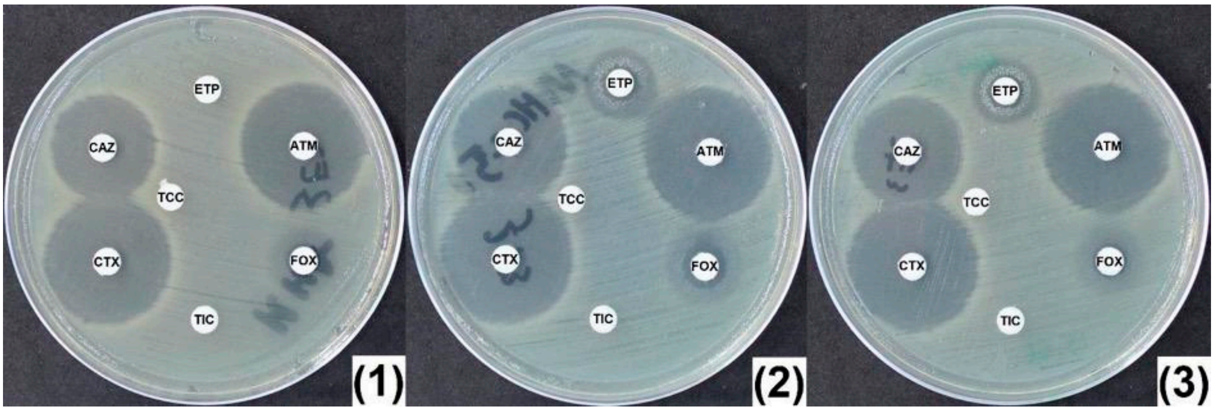

Figure S2. TEM-1B producing *E. coli*. Ertapenem MIC = 16 mg/L. A slight increase in diameters can be observed on MHC250 and MHO300 due to the natural cephalosporinase of *E. coli*, but not over the 5 mm positive threshold.
(1) Mueller-Hinton Agar (MHA); (2) Mueller-Hinton Agar supplemented with 250 mg/L cloxacillin (MHC250); (3) Mueller-Hinton Agar supplemented with 300 mg/L oxacillin (MHO300). ATM: aztreonam (30 µg); CAZ: ceftazidime (10 µg); CTX: cefotaxime (5 µg); ETP: ertapenem (10 µg); FOX: cefoxitin (30 µg); TIC: ticarcillin (75 µg); TTC: ticarcillin/clavulanic acid (75/10 µg).

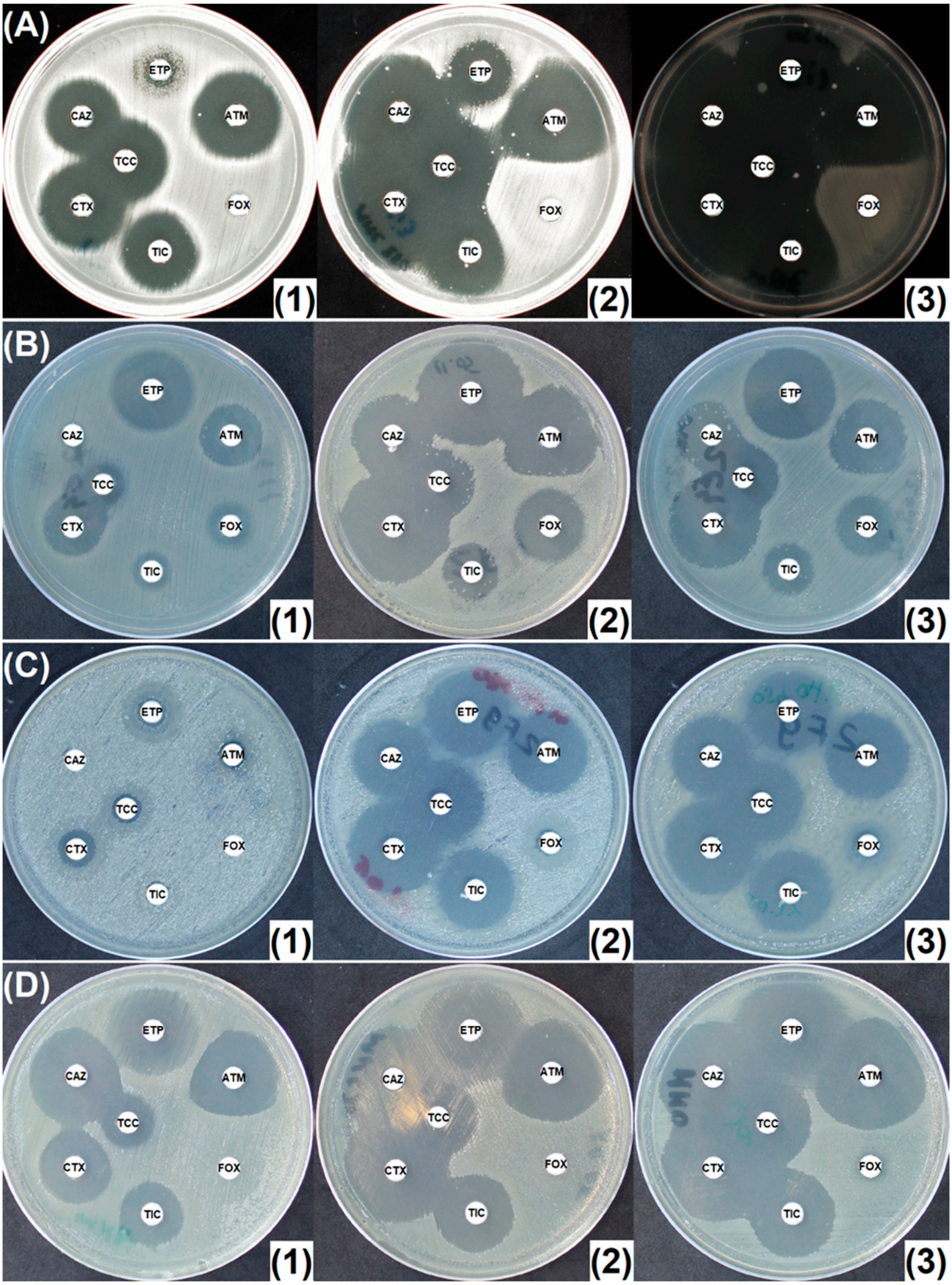

Figure S3. CASE and HCASE producing Enterobacterales with various changes in inhibition diameters. (A): IMI producing *E. cloacae* showing large inhibition diameters (> 5 mm; 11 mm increase around ETP on MHC and 23 mm around ETP on MHO); (B) *K. pneumoniae* harboring DHA-2 cephalosporinase. Larger inhibition diameters can be observed on MHC

compared to MHO, (C): *E. coli* AmpC hyper-producer; (D): *S. marcescens* AmpC hyper-producer.

(1) Mueller-Hinton Agar (MHA); (2) Mueller-Hinton Agar supplemented with 250 mg/L cloxacillin (MHC250); (3) Mueller-Hinton Agar supplemented with 300 mg/L oxacillin (MHO300). ATM: aztreonam (30 μg); CAZ: ceftazidime (10 μg); CTX: cefotaxime (5 μg); ETP: ertapenem (10 μg); FOX: cefoxitin (30 μg); TIC: ticarcillin (75 μg); TTC: ticarcillin/clavulanic acid (75/10 μg).
